# ‘It’s like trying to ice a cake that’s not been baked’: a qualitative exploration of the contextual factors associated with implementing an evidence-based information intervention for family carers at the end of life

**DOI:** 10.1017/S146342362000050X

**Published:** 2020-11-20

**Authors:** Amy Mathieson, Karen Luker, Gunn Grande

**Affiliations:** 1 Department of Public Health, Policy and Systems, University of Liverpool, Liverpool, UK; 2 Division of Nursing, Midwifery and Social Work, School of Health Sciences, The University of Manchester, Manchester, UK

**Keywords:** implementation, end-of-life care, evidence-based intervention, family carers, home care, Normalization Process Theory

## Abstract

**Aim:**

To explore the introduction of an evidence-based information intervention – the ‘Caring for Someone with Cancer’ booklet – within home care and end-of-life care, to inform future implementation and practice development within this setting.

**Background:**

Family carers’ contribution is crucial to enable care and death of people at home. The ‘Caring for Someone with Cancer’ booklet received positive responses from family carers and District Nurses and is an evidence-based intervention designed to support carers to deliver basic nursing tasks. Further feasibility work was required to establish how it should be implemented. Little is known about how to successfully translate interventions into practice, particularly within home care settings and end-of-life care.

**Methods:**

Implementation of the ‘Caring for Someone with Cancer’ booklet, utilising a qualitative case study approach, in four home care sites. Semi-structured interviews, informed by Normalization Process Theory (NPT), were undertaken at implementation sites in May 2016–June 2017. Participants were generalist and specialist nurses, managers, and Healthcare Assistants (HCAs). A framework approach to analysis was adopted.

**Findings:**

Forty-five members of staff participated. Failed implementation was associated with organisational-level characteristics and conditions, including workforce composition and predictability of processes. Unstable work environments meant home care providers focused on short-term rather than long-term goals, precluding practice development. Staff’s perceptions of the time available to engage with and implement the intervention inhibited adoption, as many participants were *“just getting through the day”*. Implementation was successful in sites with explicit management support, including proactive implementation attempts by managers, which legitimatised the change process, and if all staff groups were engaged. To encourage uptake of evidence-based interventions in home care settings, practitioners should be given opportunities to critically reflect upon taken-for-granted practices. Future implementation should focus on work pertaining to the NPT construct ‘Collective Action’, including how staff interact and build confidence in new practices.

## Background

Family carers’ presence and contribution have been widely recognized as a predictor for enabling patients’ end–of–life care at home (Gomes and Higginson, 2006). Moreover, family carers’ ability to cope with the role, specifically their ability to manage symptoms, influences the likelihood of death at home (Ullgren *et al*., 2018), whilst physical and emotional exhaustion from the burden of caregiving acts as a barrier (Wahid *et al*., 2018). It is, therefore, important that healthcare professionals try to meet family carers’ needs by providing timely, tailored support.

Evidence suggests an important component of this support is the provision of information and skills training necessary to undertake physical nursing tasks (Bee *et al*., 2009). This information could increase carers’ mastery and confidence, and carers may find it easier to accept support that enables them to provide care than to accept help for themselves (Grande *et al*., 2009).

The ‘Caring for Someone with Cancer’ booklet is an evidence-based intervention, which was developed to be used by District Nurses to support family carers to care for someone at home towards the end of life by identifying some of the skills required to complete basic nursing tasks. Guided by the Medical Research Council (MRC) framework for developing and evaluating complex interventions, the resource was developed based on existing evidence (Medical Research Council, 2008; Bee *et al*., 2009, Caress *et al*., 2009) and interviews with current and recently bereaved family carers. Findings from the feasibility study suggest the booklet has the potential to have a positive impact on family carers and may facilitate more people to die at home, by reducing uncertainty and providing reassurance. District Nurses stated they received fewer phone calls from family carers as a result, potentially reducing their workload (Luker *et al*., 2015). In addition, further work revealed the booklet has wider utility: using it for other conditions, to support relatives to be involved in the care of residents in nursing homes, and in Healthcare Assistants’ (HCAs) end-of-life care training (Mathieson, 2018). Important questions remained, however, regarding when and how to deliver the booklet, suggesting more work was required to answer how best to implement the intervention and maximise its usefulness. This was in line with the MRC Framework (2008), and contributed to the current need to translate models of support for family carers into realistic applications for practice (Ferrell and Wittenberg, 2017).

The evidence base for effective implementation^1^ of complex interventions remains limited (Medical Research Council, 2008), particularly within home care^2^ nursing (Brooke and Mallion, 2016). Implementation studies in this area are challenged by inconsistent terminology, and limited use of theory (Mathieson *et al*., 2019). Thus, there is a need to assess the extent to which implementation is effective in home care nursing. We, therefore, aimed to implement the booklet intervention in four home care provider sites.

### Normalization Process Theory

To address the gap of a limited use of theory in implementation studies in home care, Normalization Process Theory (NPT) guided this study. NPT focuses on the process through which new ways of thinking, interacting, and organising work are embedded, sustained, and ‘normalized’ in practice (May and Finch, 2009). This theory was considered the most appropriate approach to guide implementation of the intervention after conducting a review of implementation science literature, which evaluated the potential application of 48 different models, theories, and frameworks (Mathieson, 2018). The reason for using NPT was threefold. First, NPT is compatible with qualitative research and offers specific applications for researchers when designing research, collecting, and analysing data. Second, NPT is useful to explore the barriers and facilitators to embedding the intervention in practice. Third, by using NPT, it is possible to explain the extent to which the new practice has become normalised, thus evaluating the implementation efforts. Furthermore, few studies have tested its use to guide the implementation process (McEvoy *et al*., 2014). This study, therefore, aimed to test the prospective use of NPT.

NPT has four constructs, each with four corresponding components (Table 1) (May *et al*., 2015). This paper reports on data pertaining to the construct ‘Collective Action’, defined as the ‘operational work’ people do to enact new practices (May *et al*., 2015). The construct ‘Collective Action’ is therefore useful to understand successful implementation within the study’s context.

**Table 1.** Summary of Normalization Process Theory’s (NPT) constructs and components

COHERENCE: What people do to understand and conceptualise the intervention and their work
**Differentiation** What people do to understand how a set of practices and their objects are different from each other. What they do to organise the differences
**Individual specification** Individuals’ understanding of their specific tasks and responsibilities around a set of practices.
**Communal specification** People working together to build a shared understanding of the aims, objectives, and expected benefits of a set of practices. How a team works out how to integrate an innovation into their healthcare setting.
**Internalisation** Work to understand the value, benefits, and importance of a set of practices. The work people do to attribute worth to a new way of working.
**COGNITIVE PARTICIPATION: Relational work that people do to build and sustain a community of practice around a new technology or complex intervention**
**Initiation** The work people do to drive forward the new or modified practice. Setting things up and working with others to make things happen.
**Enrolment** How participants organise and reorganise themselves and others in order to collectively contribute to the work involved in new practices.
**Legitimation** Work to ensure other participants believe it was right for them to be involved, and that they could make a valid contribution.
**Activation** The work of keeping the new practices in view and connecting them with the people who need to be doing them.
**COLLECTIVE ACTION: Operational work that people do to enact a set of practices: Organisational resources, training, division of labour, confidence, and expertise as well as the workability of the intervention in clinical interaction**
**Interactional workability** The interactional work that people do with each other, with artefacts, and with other elements of a set of practices, when they seek to operationalise them in everyday settings.
**Relational integration** The knowledge work that people do to build accountability and maintain confidence in a set of practices and in each other as they use them.
**Skill set workability** Allocation work that underpins the division of labour that surrounds the new practice: who does what and how it relates to their existing skill set.
**Contextual integration** The resource work – managing a set of practices through the allocation of different kinds of resources and the execution of protocols, policies, and procedures.
**REFLEXIVE MONITORING: Appraisal and monitoring of implementation work.**
**Systematisation** Work of collecting information on how effective the intervention is for themselves and others.
**Individual appraisal** Individuals evaluating the use of the innovation in relation to their own work practice
**Communal appraisal** Participants work together – sometimes in formal collaboratives, sometimes in informal groups to evaluate the worth of a set of practices.
**Reconfiguration** The appraisal work by individuals or groups, which may lead to attempts to redefine procedures or modify practices – and even to change the shape of the innovation itself.

### Aims

The study’s aims were to: (1) actively implement the booklet intervention with home care providers using NPT and (2) identify barriers and facilitators for a successful implementation of the booklet intervention, including contextual factors that influence adoption to inform future implementation of evidence-based interventions in this setting.

## Methods

### Study design

Due to the issues raised in the feasibility study (Luker *et al*., 2015), a qualitative approach was adopted to identify ‘task’, ‘social’, and ‘physical’ context levers, which may affect implementation and adoption within the study setting (Johns, 2006). Specifically, a qualitative case study was considered a useful technique, as little was known about the phenomenon; multiple perspectives needed to be recognised; and the research needed to be congruent with clinical practice (Walshe *et al*., 2004).

We designed a qualitative case study based on Stake’s methodology (Stake, 1995). Data for this paper are drawn from semi-structured interviews with key staff, identified as gatekeepers to information, within the case study sites.

### Setting

The case was defined as ‘home care settings in one city in the North West of England actively implementing the “Caring for Someone with Cancer’ booklet”. Palliative care is provided by District Nurses in patients’ homes, by Registered General Nurses and HCAs in nursing/residential homes, and by the hospice in-patient and out-patient services (Hospice@Home and Community Specialist Palliative Care Team). Four cases (two nursing homes, a District Nurse team, and a Hospice@Home team) within the case study area were purposefully selected to actively implement, and evaluate the implementation of the intervention (Table 2).

**Table 2.** Description of case study sites

Case study site	Description of care provided	Number of staff	Caseload/number of residents	Patient/resident demographics
**1 – Nursing Home 1**	Nursing care	46 (6 RGNs^a^, 40 HCAs^b^)	35	Predominately Huntington’s disease, nultiple sclerosis, Parkinson’s disease, dementia, and cancer patients.
**2 – Nursing Home 2**	Nursing care and residential	37 (7 RGNs, 30 HCAs)	41	Ex-servicemen. Specialist care categories including hearing impairment, Huntington’s disease, motor neurone disease, Parkinson’s disease, speech impairment, stroke, and visual impairment.
**3 – District Nurse Team**	Physical care provided to people with disabilities and long-term conditions, including wound care and palliative, supportive, and end-of-life care. Promotion of psychological and social well-being.	18 (1 Team Leader (RGN), 1 District Nurse Sister (RGN), 16 District Nurses^c^ (RGN))	389	Predominately chronic obstructive pulmonary disease, Mmotor neurone disease, heart failure, and multiple sclerosis patients. Large Jewish population.
**4 – Hospice@Home Team**	Mainly psychological and emotional support for patients and family carers. Limited ‘hands-on’/physical care.	7 (1 Team Leader, 1 Staff Nurse (RGN), 1 Assistant Practitioner, 4 HCAs).	49	Predominately cancer patients and their carers. Some motor neurone disease, chronic obstructive pulmonary disease, heart failure, and Parkinson’s disease.

a RGNs or Registered General Nurses are nurses who have completed a 3-year training course in all aspects of nursing care to enable him/her to be registered with the UK Central Council for Nursing, Midwifery, and Health Visiting.

b HCAs or Healthcare Assistants provide care to patients in hospitals or nursing/residential homes (residents’ ‘usual place of residence’) under the direction of a qualified professional. HCAs are sometimes known as nursing assistants, auxiliary nurses, or support workers.

c District Nurses are RGNs with specialist practitioner qualification.

Information sessions, led by the researcher (AM), were used to ‘kick start’ implementation (Wilcox, 2010, Kapp, 2013). Information sessions lasted 30 min and provided details about the booklet’s development, study’s purpose, and expectations of the sites involved. At the sessions, the researcher explained the Internal Facilitator role, which involved becoming a voluntary member of the research team, acting as the intervention’s champion for the site, and attending regular meetings. Internal Facilitators were recruited from all sites.

### Data collection

Interviews were conducted by one researcher (AM). Topic guides were tailored to individual case study sites and were informed by NPT (Box 1) (May *et al*., 2015). Data, before and after implementation, were collected between May 2016 and June 2017. Most people (*n* = 35) took part in one interview (pre- or post-implementation). Reasons for this included work commitment, staff rostering, participants leaving the organisation, or site withdrawal from the study.

Box 1. Topic guide questions and example of questions for specific case study sites

General topics covered:

Participants’ background and work experience

The provision of information/support for relatives and participants’ end-of-life care training (*Coherence)*

Initial impressions of the intervention (*Coherence and Cognitive Participation*)

Implementation strategies, delivery processes, and implementation work (*Collective Action*)

Evaluation of the intervention and its implementation (*Reflexive Monitoring*)

Hospice@Home and District Nurses

*Pre-implementation interviews:*

Is supporting family carers an important part of your role? Why/why not?

How would you identify which family carers to give the booklet to?

How does the booklet relate to the support you are already giving family carers?

*Post-implementation interviews:*

How did you work with others to implement the booklet?

Did you have any opportunities to evaluate the usefulness of the booklet?

When might it be difficult to use the booklet? Why?

How do you feel about carrying around copies of the booklets when you visit patients and their carers?

Nursing homes

*Pre-implementation interviews:*

What end-of-life care training have you received?

What challenges have you faced when putting this training into practice?

How important is it to give relatives help with practical care needs?

How could the booklet help with explaining care and what to expect with relatives?

*Post-implementation:*

Have recent changes had an impact upon implementation of the booklet? Why/why not??

What is your understanding of the benefits of using the booklet (for staff and relatives)?

Has your understanding of your role in supporting relatives changed? Why/why not?

### Sampling and recruitment

All staff members who had access to the booklet, or could provide information about its implementation, were eligible. Internal Facilitators initially contacted potential participants who, with their permission, were introduced to the researcher. All participants had time to consider their involvement, and interviews were arranged at a time convenient to them. Snowball sampling techniques, whereby participants were asked to suggest potential informants, were used once data collection was underway (Coyne, 1997).

Interviews were held in private rooms at the case study sites. Interviews averaged 46 min in length. All interviews were digitally recorded, transcribed verbatim, and anonymised. Written informed consent was obtained from participants prior to each interview. All participants received a ‘Certificate of Participation’ in exchange for their time, which were thought to be an incentive for skilled staff to include in their professional development portfolio and/or contribute to revalidation.

Forty-three females and two males participated, spread across the four case study sites (see Table 3 for a breakdown of roles).

**Table 3.** Participants interviewed

Case study site	Participants	Total
**1**	3 Registered General Nurses 11 HCAs 1 End-of-life care Trainer 1 Activities Co-ordinator 1 Training Co-ordinator 1 Nursing Home Owner 1 Nursing Home Manager	19
**2**	3 Registered General Nurses 7 HCAs 1 Activities Co-ordinator	11
**3**	5 District Nurses 1 District Nurse Sister 1 Assistant Nurse Practitioner	7
**4**	1 Staff Nurse 3 HCAs 1 Hospice@Home Co-ordinator 1 Specialist Palliative Care Pharmacist 1 Assistant Practitioner 1 Clinical Service Manager	8

### Data analysis

Framework analysis was used to facilitate within and cross-case analysis (Ritchie and Spencer, 1994). The researcher (AM) familiarised herself with the data by reading and re-reading transcripts. Recurring ideas were collated into groups of similar concepts and organised into an index (Furber, 2010), which was used to inform open coding in NVivo 10, generating a number of thematic ‘sets’ (Spencer *et al*., 2014). The research group (AM, GG, and KL) then met to refine and collapse coding categories, and discuss any discrepancies of interpretation. Summaries of indexed extracts were developed for each thematic set to facilitate within-case analysis. Cross-case analysis was facilitated by examining similarities and differences between the underlying elements identified in the data. Table 4 summaries the analysis process.

**Table 4.** Summary of analysis process

**Phase 1**	**Familiarisation**
	Reading and re-reading transcripts and field notes. Transcripts were annotated and memos were kept. Recurrent topics or issues across the data set were identified.
**Phase 2**	**Constructing an initial framework of emerging themes**
	Recurring topics or issues were collated into groups of similar ideas and organised into an index. The index was used to inform the coding of the data directly in NVivo 10. Through the process of free coding, nodes were created in NVivo 10. The process was repeated until all segments of the text were assigned to the created nodes.
**Phase 3**	**Indexing and Sorting: Coding the data and application of the NPT framework**
	Folders were created within NVivo 10 to separate the different data sets. Interview field notes were uploaded as memos. In addition to free coding, the NPT framework was applied directly to the data. The NPT constructs were added to Nvivo 10 as the main theme, with the corresponding components as sub-theme. The data was sorted so that interview extracts exploring the same issue were grouped together. Nodes were reviewed and refined by examining the coded segments. Nodes with similar properties were merged and labels were changed, reducing the total number of nodes from 836 to 786. The data was sorted in NVivo 10 by bringing together all the data that had been indexed in the same way, creating a number of thematic ‘sets’, and a hierarchy of themes and sub-themes.
**Phase 4**	**Charting – data summary**
	Indexed extracts from the interviews were summarised.
**Phase 5**	**Synthesising Data by Mapping and Interpreting**
	Themes and sub-themes were explored using ‘mind maps’. This process facilitated the merging of themes and sub-themes, the creation of explanatory accounts, and linkage between case study sites The data was interpreted by detecting elements and underlying dimensions in each theme. The data was examined again, creating a set of categories and typologies which explained the data.

## Findings

Five themes, and corresponding sub-themes, were identified regarding the contextual factors, organisational culture, and implementation capability, which made changing practice within the sites difficult, or conversely more straightforward.

### Theme 1: Workforce and predictability of processes

Organisational changes within the NHS in general and specifically within the organisations, in conjunction with demands on staff, possibly due to competitive tender, appeared to result in a number of staff leaving across case study sites. All participants, with the exception of Hospice@Home, discussed the high turnover of staff and current ‘*unsettled’* state of the organisation: ‘*There*’*s a lot of disharmony at the moment, because of poor staffing levels and large turnover of staff’* (CHS9 Site 1). This was widely stated as the reason for failed implementation attempts, and was recognised as a potential barrier to other areas of practice development: ‘*you don*’*t want to start a research project with somebody that*’*s going to leave’* (HaH01 Site 4).

For staff in Nursing Home 1, there was a sense of uncertainty regarding its future, as the current contract for the unit that provided end-of-life care was out for tender. This had an impact on morale, and resulted in some staff seeking other employment, which inhibited implementation and adoption of the booklet. Participants expressed feelings of hopelessness and the futility of developing current practice; changes may not be sustained and the future of the unit was uncertain: ‘*I don*’*t really know how things are going to pan out in the future. It*’*s like the million dollar question’* (CHS1 Site 1). This suggests there was an absence of ‘change efficacy’; the shared belief in the staff’s collective capability to execute actions involved in implementation (Weiner, 2009). Consequently, some staff did not engage in the work required to embed the new practice.

Within the nursing home settings, staff turnover and use of agency staff – temporary or long-term workers hired from an employment agency – was widely perceived as inevitable, as it was frequently witnessed by participants. This was reflected in one participant’s understanding of her attempts to implement end-of-life care training within nursing homes:

*“I: I wonder if you’ve come across any challenges getting this [End-of-Life Care Training] into care homes and actually embedding it?*

*Absolutely, care home staff move, care home managers move, the commitment from one manager might be less than another manager, staff not being able to be released because they’re short-staffed; and what you need to remember is this is voluntary, they don’t have to take part […] so if that means that they’re leaving the residents vulnerable because there’s not enough staff on the floor then they’re not going to attend your training” – TS01*

Similarly, the District Nurse site was undergoing a turbulent period due to staff shortages. The District Nurse team was understaffed for the study’s duration and relied upon agency staff. District Nurse participants discussed the challenges associated with everyday practice, and it was considered an inappropriate time to introduce and integrate something new: ‘*I just don*’*t think at the moment the timing*’*s right to be introducing new stuff when we*’*ve got new staff coming through all the time’* (TS02 Site 3). Despite evidence to suggest the booklet intervention could save clinical time (Luker *et al*., 2015), and thus facilitate its adoption, participating nurses chose not to use it and rather focused on immediate challenges. The nursing homes and District Nurse team appeared to be ‘in limbo’, waiting for the service to ‘*settle down’* before implementing the booklet. This is in contrast to Hospice@Home site, which had a stable workforce, and successfully implemented the booklet: ‘*your HCAs, your unqualified staff, tend to stay the same. There tends to be a core of nurses’* (HaH08, site 4).

A stable workforce was thus considered an important facilitator when implementing new practice: ‘*otherwise it*’*s like trying to ice a cake that*’*s not been baked’* (TS01); with a lack of continuity inhibiting adoption.

### Theme 2: Guidance and shared vision

#### Support from colleagues

The availability of support from other staff members was a key factor in ensuring the successful implementation of the booklet, particularly within the nursing home settings. Specifically, the lack of continuity with nurses in this setting, who were perceived as leading the intervention, and the reliance on agency nurses, resulted in individual adoption, which was haphazard:

*“ We’ve had different nurses…when [name of nurses] were here they’d say “I’ve just given such and such that book”…the [nurses] we’re getting now, they’re not here on a regular basis…the one we have on today might not be on for another couple of weeks… so it’s not the same communication”* - *CHS3, Site 1*

When reflecting upon past practice development, many participants discussed the lack of direction from managers and few allocated resources to support staff adjusting to change, resulting in staff feeling ‘abandoned’ and ‘alienated’. Participants claimed they were ‘*upset’* and ‘*disheartened’* by the absence of manager support, suggesting there was a disconnect between the manager’s perception of the change process and staff’s morale, and that belonging to frontline staff. This often resulted in staff leaving the organisation, which had an impact on subsequent practice development:

*“If I was the owner of a factory and suddenly all my senior staff or very experienced carers were upping and leaving, I would have been down on the shop floor saying “what’s going on?”, and that’s what we didn’t get. And it was soul destroying.” – CHS1, Site 1*

#### Communication

Staff’s sense of ‘abandonment’ was exacerbated if the changes were perceived to be ‘*sweeping’*, or if there was a lack of communication from managers regarding developments. For example, HCAs in nursing home settings usually became aware of changes to practice through ‘word of mouth’: ‘*One day when you’re off it just comes, this is a new thing, you have to go and do it’* (CHS204 Site 2). Thus, HCAs appeared to be ‘kept in the dark’ regarding current or future changes. Conversely, nurses were informed of practice developments, which was then cascaded down to HCAs:

*“I: When something new is introduced, how are you told about it?*

*Email or you get told by the nurses. They get the information first and they pass it on to us.” – CHS209, Site 2*

New practices were therefore largely adopted and normalised by nurses, whereas HCAs’ adoption was often delayed.

### Theme 3: Management of time

Lack of time and large caseloads was a frequently reported barrier to the engagement with the booklet intervention. District Nurses claimed it was difficult to ‘*do anything extra’* as they were ‘*just getting through the day’*; thus, prioritising other tasks over implementation work. However, the time required to deliver the booklet was minimal, suggesting the reported lack of time was instead a consequence of family carers having more information and thus asking nurses more questions. By claiming to ‘be busy’, nurses may restrict patients’ and family carers’ ability to ask for, or access, additional support (Nagington *et al*., 2013). Furthermore, some nurses were convinced they ‘knew’ so-called ‘tricky’ relatives would ‘*tick off’* symptoms in the booklet and apply them to the patient/resident, which could cause upset and generate more work for nurses.

Staff also claimed they required time to become familiar with the intervention, which was considered as an essential preparation work to build accountability and confidence in the new practice (May *et al*., 2015). Many District Nurse participants claimed they did not have time to engage in this ‘preparation work’, inhibiting their adoption. Moreover, District Nurses did not have the time to critically reflect upon their practice and identify how the booklet could be integrated. Value, therefore, was not attributed to the booklet, nor was the intervention’s potential to save time realised.

Additionally, as District Nurses anticipated delivering the booklet would add extra time to an already busy visit, and not wanting to overburden staff, the Internal Facilitator did not champion the booklet nor reminded staff to use it. Regardless of initial buy-in from the Internal Facilitator and management endorsement, workload and a perceived lack of time were, therefore, too much of a barrier for many District Nurse participants:

*“…I’ll be brutally honest with you, when you’re sitting here in a huddle and you’re saying “this is what I’m doing and I need you all to go out and do this [deliver the booklet]” they’re not thinking about that, they’re thinking about their next visit because they’re busy…” – TS02, Site 3*

Similarly, in the nursing homes, participants discussed the chaotic nature of their work, claiming there was ‘*never a dull moment’* (CHS1 Site 1) and there was ‘*never a break’* (CHS9 Site 1). One participant compared her increasing workload to being on a ‘rollercoaster’, unable to fulfil all her duties within the time available during a shift. Consequently, nursing home staff had to prioritise their workload. Nursing home participants claimed residents care ‘comes first’. Therefore, work related to the resident’s care including new admissions, death of a resident, and communication with relatives was perceived as tasks that required immediate action and were thus prioritised, delaying implementation work.

In addition, scheduled tasks within the nursing home settings took priority. For example, medication rounds consumed the majority of nurses’ time and could be considered a ‘scheduled task’, as they occur at the same time every day. Documentation presents another demand on nurses’ time, which precluded their involvement in implementation and practice development. Documentation makes the invisible visible, which arguably improves communication and continuity of care (Gjevjon and Hellesø, 2010), but also places staff under surveillance, possibly accounting for why staff spent time on it. Completing documents could be perceived as ‘maintenance tasks’, which were continuous throughout the day and had an impact on other areas of their practice. Specifically, due to the ‘*demands of modern auditing’* (CHS1 Site 1), which was compounded by staff shortages and the use of agency staff, nurses were unable to interact with relatives, which may involve delivering the booklet.

### Theme 4: Organisational ethos, leadership, and culture

Successful implementation required the organisation to be conducive to change, whereby sufficient resources to support the change process were allocated. Across the sites, a variety of perspectives were expressed regarding research and changing practice. Some participants emphasised the need to ‘*go with the times’*, whereas others claimed they preferred ‘*old school nursing’* (CHS202), limiting their engagement in practice development. Overall, the hospice, in which the Hospice@Home team was based, was perceived as ‘*an open culture for change’*, which aimed to ‘*develop and share’.* Consequently, Hospice@Home staff were given time to participate in the research and were encouraged by the manager. It is likely that Hospice@Home participants had ‘*more time’* to engage in implementation work compared to other sites, most notably District Nurses.

Leadership was considered a crucial factor in potential adopters gaining the confidence to use the intervention. For example, in Nursing Home 1, there were persistent claims that implementation ‘*fell by the wayside’* because the two Internal Facilitators left, who were perceived as influential local champions. Leadership, including the influence from senior nurses as role models, therefore facilitated the implementation of the booklet intervention.

In contrast, HCAs in the Hospice@Home team had clear leadership and role models in the service co-ordinator and staff nurse. Participants perceived the Hospice@Home Internal Facilitator as the ‘*hub’*, who engaged in work to keep the intervention visible. In turn, the Clinical Service Manager supported the Hospice@Home Coordinator in her role as Internal Facilitator, describing herself as a ‘*leader’* rather than a manager who is ‘*more hands on than most people’* (HaH08 Site 4). Participants suggested that the characteristics of a ‘good leader’ includes being knowledgeable of patients, residents, and their families; and taking a ‘*hands on’* approach yet giving staff the freedom to organise and develop their practice. Furthermore, constantly communicating with staff, being a ‘*port of call’* and approachable were perceived as characteristics of a ‘good leader’. The abovementioned ‘good leader’ was a facilitator in this study.

### Theme 5: Staff’s understanding of the innovation and its workability

#### Applicability

As end-of-life care was the main remit for the Hospice@Home team, the intervention was considered relevant to staff within this site, and thus potential adopters perceived engagement in implementation work to be a legitimate use of their time. The team was, therefore, open to the change process and the booklet was successfully integrated into their routine practice. In contrast, some participants at other sites – where end-of-life care was only a partial remit – considered the innovation to be incompatible with their norms, values, and perceived needs of family carers. These participants resisted adoption and did not deliver the booklet.

#### Adaptability

The Hospice@Home participants also demonstrated that they worked as a team, and staff felt supported by their colleagues. In contrast to the nursing home sites, there was a sense amongst the Hospice@Home participants that each member positively contributed to the team’s work, and the booklet’s implementation. Specifically, participants discussed the knowledge and expertise within the team, which was accessed when supporting family carers. Consequently, not only was the booklet intervention applicable for staff within this site, but also staff were able to adapt the innovation by providing additional support – with help from colleagues – to meet the needs of their service users; facilitating implementation and encouraging adoption.

HCAs within the nursing home settings claimed they did not feel confident delivering the booklet, as they may disclose prognosis or may be unable to answer relatives’ questions, indicating a ‘Closed Awareness’ (Glaser and Strauss, 1965). Conversely, Hospice@Home HCAs felt valued, supported, and were able to use the intervention. It is likely that this respect amongst staff, sharing of expertise, and acknowledgment of individual capabilities, gave Hospice@Home HCAs the confidence to trial and adopt the booklet.

Hospice@Home staff, compared to HCAs within the nursing home settings, were autonomous; not only in the management of their caseload but also within practice development, including the adoption of the booklet. According to one participant, nursing home HCAs required ‘*discipline’* (CHS10); indeed, they needed to be ‘told what to do’. This suggests leadership is not only needed to support staff adjusting to change, but is also important in giving potential adopters the authority to change their practice. That is, not the autonomy to introduce changes without approval from the manager, rather directing staff on how to change their practice based on decisions they have made:

*“I think… giving out these books, we can’t do that. We need our management to tell us, we can do it” – CHS207*

## Discussion

In this study, we aimed to better understand the implementation of evidence-based interventions in home care settings by exploring adopters’ experience of implementing the ‘Caring for Someone with Cancer’ booklet in four case study sites. We identified a number of contextual factors, which inhibited or enabled practice development in home care settings, including workforce composition and predictability of processes; management and peer support; time management; and organisational ethos, leadership, and culture.

‘Context’ is widely recognised as a contributing factor to implementation in the theoretical literature (Damschroder *et al*., 2009; Weiner, 2009; May *et al*., 2016), including the well-known distinction between ‘receptive’ and ‘non-receptive’ contexts for change (Pettigrew, 1985). Features of organisational context in this study, which dictated the support for the use of the intervention, included communication; leadership; learning culture; and the intervention’s compatibility with, and relevance to, individuals’ work. Implementation was also affected by broader economic and social contexts (‘outer’ context) (Damschroder *et al*., 2009), including a national shortage of nurses (NMC, 2017) and HCAs (Skills for Care, 2017), and low morale amongst District Nurses (Drennan, 2019). This may be because people are being discharged ‘quicker and sicker’ from the hospital, which is having a detrimental effect on the workload of District Nurses (Speed and Luker, 2004). Whilst this pattern has existed for over 15 years, it is arguable this is increasing due to the current shortages of hospital beds. Moreover, it is a global fact that the world’s population is increasing and most countries have an ageing population (World Health Organisation, 2015), with many patients living with multiple long-term conditions (Costello, 2017). Demographic changes are therefore putting pressure on community-based care providers. This ‘outer’ context may have made otherwise receptive contexts and/or innovating individuals/groups non-receptive. In this study, due to unstable work environments, there was often an absence of ‘change efficacy’ – confidence that collectively staff can implement the change effectively – and ‘change commitment’, which inhibited implementation and adoption (Weiner, 2009). Non-receptive contexts therefore dismiss development, and foster environments in which individuals resist change.

It is widely recognised that ‘context’ is a dynamic process (Bate, 2014). However, whilst theory has identified context as having an impact upon implementation, ‘timing’ is not explicitly addressed (Nilsen *et al*., 2018), nor does the literature on implementation in primary care acknowledge that barriers may change (Lau *et al*., 2015). ‘Timing’ was identified as a significant indicator for the success or not of implementation in this study. That is, the setting in which implementation is to take place maybe, or may become, non-conducive to change, which will have an impact upon the receptiveness of individuals’ to engage in practice development (Weiner, 2009). Implementation efforts during periods of ‘bad timing’ may therefore be destined to fail, as it is like ‘*trying to ice a cake that hasn*’*t been baked’*. Thus, it is necessary to assess the context, and possibly wait for it to change, or shift the focus of the intervention (May *et al*., 2016). The work of Diffin *et al*. (2018) supports this view, and highlights the importance of assessing organisational context when implementing evidence-based intervention and planning for practitioners’ disengagement. A companion paper also highlights the importance of the facilitator role in terms of ‘leverage’ within the team, communication skills and styles, and supporting peers (Diffin *et al*., 2018a). In the current study, staff required support; staff levels needed to improve and new staff had to ‘settle in’; to continue with the metaphor: ‘*bake the cake and let it cool before icing’*. This finding is consistent with those of Nilsen *et al*. (2018) who found that forward-thinking coordination of change is required to successfully implement change within nursing homes. To assist with this ‘forward-thinking’, heuristics for assessing organisational readiness have been developed (Scaccia *et al*., 2015; Sharpe *et al*., 2018). Further to these recommendations, practitioners and researchers should consider ‘timing’; whether staff are focusing on other changes, or a high staff turnover, and the relevance of the intervention to potential adopters’ work, which may dictate how much time is given to its implementation.

The NPT constructs ‘Collective Action’ explained why some staff did not adopt the booklet or engage in work required to embed the new practice, as it accounts for the lack of resources invested in the management of the implementation (May *et al*., 2015). The concept ‘change fatigue’, defined as a passive resignation towards organisational change and thus a sense of apathy amongst individuals (Nilsen *et al*., 2018), may also explain why some nurses chose not to adopt the intervention. Care in the community appeared to be characterised by constant change, and consequently, staff may have had a negative attitude towards changes, including integration of the booklet. This idea is corroborated by Taylor *et al*. (2015) who found that new telehealth technologies were perceived to be a ‘fad’ by nurses because of their constantly changing practice, and were not adopted. ‘Change fatigue’ in the current study was signalled by, although may not be synonymous with, the participants’ preoccupation with short-term rather than long-term goals. Therefore, rather than a sense of apathy, being ‘tired’ of constantly changing their practice may lead to practitioners’ resisting further changes.

It has been recognised for over 15 years that management support is particularly important to the success of change processes, as managers often dictate how resources in an organisation are used (Bryar and Bannigan, 2003). Recent research has shown this continues to be a significant factor in the implementation of evidence-based practice in home care (Johnston *et al*., 2016), which was confirmed in this study. All staff should be involved in the change process from the outset. Specifically, HCAs are the main workforce and need to be harnessed and involved in the mainstreaming of interventions in this setting. To encourage HCAs’ participation in practice development, this staff group requires a ‘good leader’; practical know-how should be shared, and the contribution of individual staff members recognised. Moreover, as this study, and Nilsen’s *et al*. (2018) research has shown, leadership in the form of registered nurses as role models, can facilitate HCAs’ learning regarding the principles of palliative care and the development of knowledge and/or practice.

Competing workload was a frequently reported barrier in this study. One solution may be to offer staff dedicated time, away from clinical duties, to engage in practice development. This could reduce distractions (‘tasks that required immediate action’; ‘scheduled tasks’; and ‘maintenance tasks’) and encourage engagement. However, research suggests that setting time aside alone is not adequate (Mallion and Brooke, 2016; Renolen *et al*., 2018). Rather, our findings suggest staff actually require time to critically reflect upon their current practice. This could occur in groups (‘Communal Appraisal’), or individually (‘Individual Appraisal’) (May *et al*., 2015), but may only be possible if staff levels improve.

## Strengths and limitations of the study

Qualitative case study provided an ideal methodology, allowing the holistic exploration of interactions and contextual features of implementation. Nonetheless, our study’s conclusions were made from contact with a limited number of participants. The follow-up period (post-implementation) was also relatively limited (6–12 months). Furthermore, only 10 participants across the four sites took part in both stages: pre- and post-implementation interviews. A future study could explore the sustainability of the booklet intervention, and further test the facilitators identified in this study, to encourage the uptake of other evidence-based interventions in home care settings.

Notwithstanding the study’s limits, the findings make an important contribution to implementation literature, both within the area of home and end-of-life care as: (a) it is one of the first to use NPT to drive the implementation process, (b) provides an in-depth exploration of implementation, solely utilising qualitative methods, and (c) provides evidence that, despite the booklet being originally developed to be used as an adjunct to district nursing, it could be used within nursing home settings, both to support relatives and educate staff members. Furthermore, by exploring the delivery – or not – of the booklet, this study provided further justification for use of the intervention in palliative home care for cancer patients, and revealed with a ‘*few tweaks’* the booklet would be suitable for any life-limiting conditions. Specifically, participants that attributed worth to the intervention acknowledged there was a gap in service provision and family carers require more support to fulfil patients’ preferences to be cared for and die at home. The booklet was considered a means to pass on some of the knowledge required to fulfil this; specifically, on ‘what to expect’ when the patient approaches the end of life. The intervention was received positively by family carers across all sites, which encouraged many members of staff to use it.

## Conclusion

This study reveals unique challenges experienced by home care providers and researchers when attempting to change practice within this setting, including turbulent change and a focus on short-term rather than long-term goals. At the time of this study, care in the community appeared to be in crisis, as a result of underfunding and high staff turnover (Drennan, 2019). Participating sites were therefore not ‘ready’ for the change process and were widely under-resourced, highlighting the importance of timing when introducing change. It is likely that the challenges experienced by the researcher will be faced by others for the foreseeable future. Our findings suggest management leadership, staff involvement from the outset, and dedicated time to critically reflect upon existing and new practices may encourage future engagement with, and adoption of, evidence-based interventions in home care settings.

## Footnotes

1 For the purpose of this study, ‘implementation’ is defined as: ‘… [The] active and planned efforts to mainstream an innovation within an organisation’ (Greenhalgh *et al*., 2004, p. 582).

2 In this study, the term ‘home care’ was used to denote care in private dwellings by National Health Service (NHS) staff and care within residential and nursing homes.
